# 296. Molecular epidemiology and clinical features of pneumococcal pneumonia at a university hospital in Japan

**DOI:** 10.1093/ofid/ofad500.368

**Published:** 2023-11-27

**Authors:** Takumi Nakao, Kosuke Kosai, Norihiko Akamatsu, Kenji Ota, Fujiko Mitsumoto-Kaseida, Katsunori Yanagihara, Koichi Izumikawa, Hiroshi Mukae

**Affiliations:** Nagasaki University Graduate School of Biomedical Sciences, Nagasaki, Nagasaki, Japan; Nagasaki University, Nagasaki, Nagasaki, Japan; Nagasaki University Hospital, Nagasaki, Nagasaki, Japan; Nagasaki University, Nagasaki, Nagasaki, Japan; Nagasaki University Graduate School of Biomedical Sciences, Nagasaki, Nagasaki, Japan; Nagasaki University, Nagasaki, Nagasaki, Japan; Nagasaki University, Nagasaki, Nagasaki, Japan; Nagasaki University, Nagasaki, Nagasaki, Japan

## Abstract

**Background:**

*Streptococcus pneumoniae* is a major causative pathogen of pneumonia. In Japan, there are several concerns, such as increase of antimicrobial resistance, especially macrolide resistance, and non-vaccine serotypes. In this study, we retrospectively investigated clinical and microbiological characteristics of pneumococcal pneumonia in a Japanese university hospital.

**Methods:**

We collected clinical information of patients with pneumococcal pneumonia and examined microbiological characteristics of isolates, including antimicrobial susceptibility, macrolide resistance genes (*mefA* and *ermB*), serotypes, and multilocus sequence typing (MLST). The study included the data from 2011 to 2020 in Nagasaki University Hospital.

**Results:**

Among 435 pneumococcal isolates during the study, we analyzed 42, which were available and caused pneumonia in patients aged 15 years or older. Of the 42 patients, 28 (66.7%) showed moderate to severe severity according to the A-DROP severity scoring system, and the 30-day mortality was 4.8% (2 patients). The minimum inhibitory concentrations (MICs) of isolates were presented in Tables 1 and 2. In microbiological analysis, only one isolate (2.4%) showed penicillin resistance. Conversely, erythromycin, clarithromycin, and azithromycin resistance were observed in 37 isolates (88.1%) each. Particularly, isolates harboring *ermB* showed high-level macrolide resistance. The MICs of solithromycin, which is a newly developed fluoroketolide, was low (≤ 0.25 μg/mL) in all isolates. With regard to fluoroquinolone, levofloxacin and moxifloxacin resistance were found in three isolates (7.1%) each. The MICs of lascufloxacin, which was newly developed in Japan, tended to be lower than those of levofloxacin and moxifloxacin. Serotype 19 was the most frequent serotype (seven isolates), especially in *mefA*-positives (six isolates). The most prevalent STs were ST2331 and ST 338 (three isolates each), which were found in *mefA*- and *ermB*-positive isolates, respectively.

**Conclusion:**

Our results revealed molecular epidemiology and clinical features of pneumococcal pneumonia in our hospital. New antimicrobial agents might be useful for the treatment of pneumococcal pneumonia, including those caused by macrolide-resistant strains.

**Disclosures:**

**Takumi Nakao, MD**, FUJIFILM Toyama Chemical Co., Ltd.: Commissioned research|KYORIN Pharmaceutical Co.,Ltd.: Commissioned research **Kosuke Kosai, M.D, Ph.D.**, FUJIFILM Toyama Chemical Co., Ltd.: Commissioned research|KYORIN Pharmaceutical Co.,Ltd.: Commissioned research **Katsunori Yanagihara, MD, PhD**, FUJIFILM Toyama Chemical Co., Ltd.: Commissioned research|KYORIN Pharmaceutical Co.,Ltd.: Commissioned research **Koichi Izumikawa, M.D, Ph.D.**, Asahi Kasei Pharma Corporation: Grant/Research Support|Asahi Kasei Pharma Corporation: Honoraria|Astellas Pharma Inc.: Honoraria|DAIICHI SANKYO COMPANY, LIMITED: Grant/Research Support|DAIICHI SANKYO COMPANY, LIMITED: Honoraria|KYORIN Pharmaceutical Co.,Ltd.: Honoraria|Merck & Co., Inc.: Honoraria|Pfizer Japan Inc.: Honoraria|Shionogi & Co., Ltd.: Grant/Research Support|Shionogi & Co., Ltd.: Honoraria|Sumitomo Pharma Co., Ltd.: Grant/Research Support|Sumitomo Pharma Co., Ltd.: Honoraria|TAIHO PHARMACEUTICAL CO., LTD.: Grant/Research Support

